# Imaging and neuropathological findings in patients with Post COVID-19 Neurological Syndrome—A review

**DOI:** 10.3389/fneur.2023.1136348

**Published:** 2023-02-09

**Authors:** Jakub Okrzeja, Adam Garkowski, Bożena Kubas, Anna Moniuszko-Malinowska

**Affiliations:** ^1^Medical University of Białystok, Białystok, Poland; ^2^Department of Radiology, Medical University of Białystok, Białystok, Poland; ^3^Department of Infectious Diseases and Neuroinfections, Medical University of Białystok, Białystok, Poland

**Keywords:** Post COVID-19 syndrome, Post COVID-19 Neurological Syndrome, COVID-19, imaging, magnetic resonance imaging, computed tomography, positron emission tomography

## Abstract

Post COVID-19 syndrome is determined as signs and symptoms that appear during or after an infection consistent with SARS-CoV-2 disease, persist for more than 12 weeks and are not explained by an alternative diagnosis. This review presents the neuropathological findings and imaging findings in Post COVID-19 Neurological Syndrome: the focal point is on the manifestations of involvement evident on brain and spine imaging.

## Introduction

The quickly developing coronavirus disease 2019 (COVID-19) pandemic was caused by the severe acute respiratory syndrome coronavirus 2 (SARS-CoV-2) (1). Furthermore, the first cases of this illness were reported in Wuhan, Hubei Province, China (2). SARS-CoV-2 is typically connected with pulmonary infection which results in pneumonia, but recent studies indicate that other organs may be affected e.g., in the cardiovascular, immune, gastrointestinal and nervous systems (3).

Due to its strong affinity for the human angiotensin-converting enzyme 2 (ACE2) receptor, SARS-CoV-2 might infect the nervous system directly. This receptor is also present in neuronal and glial cells which might explain the observed neurological symptoms including: anosmia, peripheral neuropathy, and cerebrum disorders. In post-mortem studies, particles of SARS-CoV-2 were identified in the cerebrospinal fluid (CSF) and in the cytoplasm of hypothalamus and neocortex cells. Other findings in post-mortem studies were e.g., neuronal degeneration and death, oedema, cellular infiltration, and hyperplasia of the glial cells. An animal model research revealed that SARS-CoV-2 enters the central nervous system (CNS) *via* the olfactory bulb, expanding to nearby regions of the brain and causing significant perivascular inflammatory reaction and meningitis (4).

## Case definitions

Post COVID-19 syndrome is defined by the World Health Organization (WHO) as “condition which occurs in individuals with a history of probable or confirmed SARS CoV-2 infection, usually 3 months from the onset of COVID-19 with symptoms and that last for at least 2 months and cannot be explained by an alternative diagnosis” (5).

Furthermore, it has been suggested that in treated individuals, coronavirus stays latent in the CNS for an extended period of time, capable of reactivating and causing neurological problems (4). Post COVID-19 Neurological Syndrome (PCNS) might comprise symptoms associated to residual inflammatory reaction, organ failure, and the influence on pre-existing diseases (6). Secondary hypoxia, cytokine-related dysfunction, and retrograde transit *via* the olfactory nerve and bulb are factors which could cause reactivation of SARS-CoV-2 (7).

Another point is that the pandemic numbers underline the importance of thorough and constant follow-up of all people with COVID-19, including those who are initially asymptomatic patients in the acute stage of the disease, with routine screening for possible long-term neurological effects. Such situations also need continuous contact between primary care physicians and neurologists in order to appropriately document and analyze them (8). A literature search was carried out in PubMed for this review to identify studies that included neuroimaging and neuropathological examinations of patients with PCNS. The keywords used to carry out this search were: (1) “COVID-19,” “Post COVID-19,” “Post COVID-19 syndrome,” and “Post COVID-19 Neurological Syndrome” for the PCNS; (2) “neuropathology,” “autopsy,” “post-mortem,” “neuropathological,” “neuroimaging,” “brain,” “MRI,” “magnetic resonance imaging,” “PET,” “positron emission tomography,” “neuroradiology,” and “vasculitis” for the neuroimaging/neuropathological findings. All article entries resulting from the initial search were screened to identify publications reporting original data, with no restrictions on the type of manuscript. The objective of this review is to describe radiological features which can be found in many cases of PCNS. It might be helpful for physicians and neurologists diagnosing PCNS.

## Neuropathological findings after COVID-19

**Figure 1:**
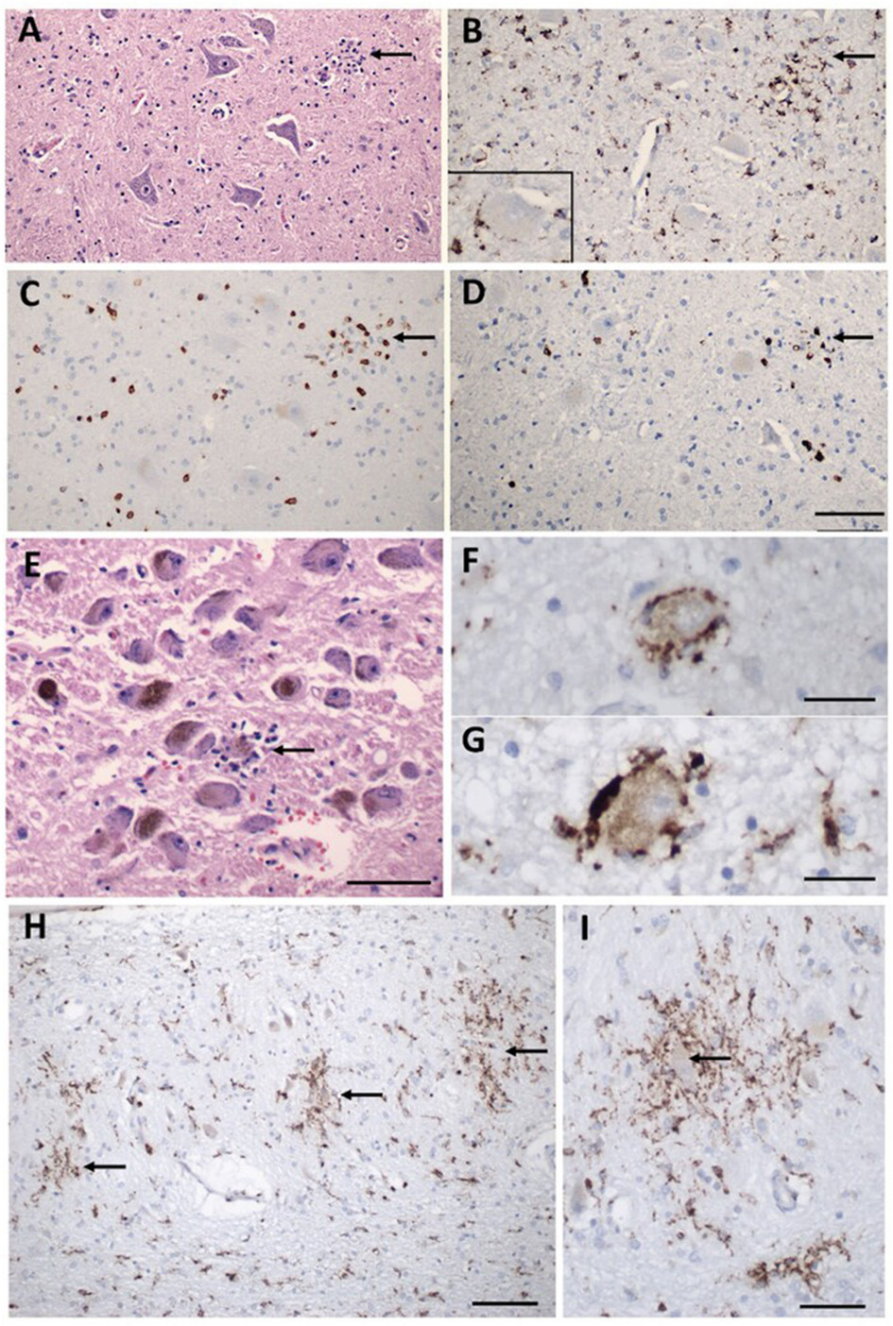
Inflammatory pathology in COVID-19 brains. **(A)** Section of the hypoglossal nucleus shows several motor neurons and a microglial module (*arrow*). **(B)** An adjacent section stained for CD68, showing clustered microglia in the nodule. Inset: Microglia in close apposition to a hypoglossal neuron (CD68). **(C)** An adjacent section stained for CD3, showing scattered T cells in the tissue and associated with the microglial nodule. **(D)** An adjacent section stained for CD8 showing that many of the T cells are CD8 +. **(E)** The locus coeruleus contains a microglial nodule with a degenerating neuron in the center, identified by its residual neuromelanin (*arrow*). **(F, G)** Neurons of the dorsal motor nucleus of the vagus surrounded by CD68 + microglia. **(H, I)** Microglial nodules in the dentate nucleus (*arrows in* **H**), neuron in the middle of a nodule (*arrow in* **I**), CD68. Scale bar in **(D)** = 200 μm for **(A–D)**; in **(E)** = 10 μm; **(F**, **G)** = 50 μm; **(H)** = 100 μm; **(I)** = 50 μm. Adapted from Thakur et al. (9) (License Number 5472500477340).

The most common neuropathologies of brain discovered in COVID-19 patients' autopsies are dispersed ischaemic/hypoxic damage, acute and subacute infarcts which could be big or slight—sometimes with a hemorrhagic constituent, vascular congestion that might be related to the hemodynamic lesions caused by the infection, and dispersed and focal microglial activation with destruction of neurons by phagocytic cells mainly localized in the lower part of brainstem (9, 10). It is not certain that all these lesions were caused by SARS-CoV-2, because some of the individuals were chronically ill and it is impossible to confirm whether all examined patients died of COVID-19 (9). Furthermore, neuronal injury and death in the lower part of brainstem might result in a variety of clinical manifestations, such as: abnormal cardiorespiratory regulation, lethargy, insomnia, and other clinical signs (9). Therefore, it is probable that associations between neurons and microglia were rather secondary to hypoxic/ischaemic damage, in the context of a systemic inflammatory response, than to a direct reaction to SARS-CoV-2 infection of nerve cells (9). Moreover, there was little T cell infiltration and no indication of acute vascular wall injury (9) (Figure 1).

The ability of SARS-CoV-2 to infect and replicate within the human brain has been demonstrated beyond a shadow of a doubt by the detection of genomic RNA and subgenomic RNA through polymerase chain reaction (PCR), numerous imaging methods presenting SARS-CoV-2 RNA and protein within cells of the CNS, and sequencing in the CNS (10) but quantitative real time-PCR (RT-PCR) on multiple frozen cerebral samples from many cerebrums revealed low or undetectable amounts of RNA of SARS-CoV-2. While there is significant heterogeneity among brain regions, the comparatively low concentrations of viral RNA imply that SARS-CoV-2 has weak CNS tropism (9). Interestingly, studies have shown that SARS-CoV-2 can occur in most regions of the nervous system, including both hemispheres, brainstem, thalamus, sciatic nerves, except the dura mater (9, 10).

## Neurological and neuropsychiatric manifestations of Post COVID-19 Neurological Syndrome

The impact of the acute phase of COVID-19 on the nervous system should be discussed to highlight the differences between acute phase of COVID-19 and PCNS. Even though some neurological consequences in COVID-19 patients have been found, the exact association between the infection and the nervous system diseases is unclear. In clinical studies conducted in Wuhan (China) 36.4% of the COVID-19 patients had CNS symptoms and 8.9% had peripheral nervous system (PNS) symptoms (11). Neurological symptoms may not usually indicate a direct infection of the CNS or PNS, but can be the result of a strong systemic response to a COVID-19 outside the CNS or PNS. Nevertheless, recently reported cases of meningitis and encephalitis, associated with the coronavirus illness, imply that SARS-CoV-2 can directly attack the neurological system (12). Acute COVID-19 manifestations such as pain in muscles, vertigo, headaches, and disorders of concentration may have a neurological cause and continue after the acute period (13, 14). Furthermore, small emboli in cerebrum (15, 16), blood-brain barrier (BBB) failure (17, 18), inflammatory reactions in CNS (19) resulting in coagulopathy, and hospital admission initiators (e.g., mechanical ventilation and sedatives) may all have a role in long-term neurological issues (19, 20).

Post COVID-19 syndrome includes psychiatric problems caused by social isolation, panic, and the loss of family members (20). The hospitalization and intensive care unit (ICU) stay, as well as the duration of critical disease, are likely to impact the occurrence of neuropsychiatric disorders after infection too (18). Moreover, chronic symptoms can be caused by a mix of physiological factors. Coronavirus RNA, for instance, might persist in cerebrum tissue for an extended period of time, exacerbating neurodegeneration (14, 21–23). Furthermore, innate immune cell infiltration associated with BBB failure may extend neuroinflammatory processes (21, 24). In addition, injury during acute illness, and chronic exhaustion are strongly linked to the development of neuropsychiatric disorders after infection (mainly sleep difficulties) (25).

The most common signs of neurological and neuropsychiatric Post COVID-19 syndrome are exhaustion, cognitive disorders such as brain fog, memory difficulties, difficulty concentrating, and sleep abnormalities which might be found in nearly one-third of individuals 12 weeks following the beginning of acute COVID-19 disease. Moreover, these symptoms remains and are much more prevalent in the short period (3–6 months) than in the long period of time (above half year after infection) (20, 26).

Furthermore, atrophy of the hippocampus and cortex (23, 27, 28), ischemic alterations (29), and small vessel disease (SVD) (30) have been demonstrated to develop as a result of inflammatory reactions and oxidative stress during COVID-19 (23, 31). The long-term effects of these processes might show as cognitive impairment e.g., brain fog, memory difficulties, difficulty concentrating.

Anosmia, i.e., loss of the sense of smell, dysgeusia, i.e., taste disturbance, and headaches are prevalent symptoms of acute COVID-19 disease, but they are not significant features of PCNS (suggesting that these particular manifestations often disappear). In a retrospective study, individuals who experienced anosmia or taste disturbance during acute coronavirus illness, 68% regained their sense of smell and above 70% regained taste after almost 2 months from the beginning of the symptoms (32).

Less common disorders in PCNS are: Guillain-Barré syndrome (GBS), polyneuropathy, myopathy, encephalopathy, post-infectious transverse myelitis, seizures, parkinsonism, orthostatic hypotension which is connected with vasovagal syncope, strokes and neuro-ophthalmology problems, i.e., post-infectious optic neuritis—these disorders have been found in some of the individuals at the 12 weeks follow-up (33).

GBS has been more common since the epidemic began. Some papers have been published showing the link between SARS-CoV-2 infection and GBS (34). The great majority of GBS patients with COVID-19 have been para infectious, whereas GBS after infection is rare (35). There were twelve GBS cases described after healing from COVID-19. In this context, there is increased interest in the connection between COVID-19 and the progression of GBS which typically occurs during the early stages of infection (36). Several hypotheses have been proposed to describe the genesis of GBS following COVID-19. The most likely mechanism is the production of antibodies against SARS-CoV-2 surface glycoproteins which might induce peripheral nerve damage due to similar native protein forms (34).

Consistent with previous studies, the chance of cerebrovascular episodes rose after COVID-19, with the frequency of ischemic stroke rising to roughly 1 in 10 (20). SARS-CoV-2 may cause stroke by a number of processes, such as: invasion of the vessel walls which results in coagulopathy due to endothelial inflammatory reactions, heart damage which results in clot formation, or destabilization of an atheroma plaques (37). Stroke has been observed in multiple studies about active COVID-19 disease, but stroke following COVID-19 without active illness has only been described in a few clinical studies (38).

According to analyses on SARS-CoV-1 and MERS, the majority of individuals return to full health after these viral diseases and might have many neurological sequelae even years later (39). Despite extensive studies on COVID-19 neurological symptoms and sequelae, only a few examples of neurological effects after full recovery from COVID-19 illness have been reported (40).

## Which radiological features can we find in the imaging of the Post COVID-19 Neurological Syndrome?

Radiological examinations are one of the most important aspects of the diagnosis of many diseases. They are very often needed by doctors to confirm their current diagnostic hypotheses. PCNS is a relatively new neurological disorder, so only few studies describe the radiological imaging in the PCNS. This review brings together the radiology achievements of the PCNS imaging. The most useful types of imaging of the PCNS are magnetic resonance imaging (MRI), positron emission tomography/computed tomography (PET/CT) and computed tomography (CT).

The capacity to perform a whole-body examination, good individual compliance, and a high safety profile are benefits of PET/CT (41). The brain 18F-FDG-PET signal was taken as a direct indicator of neuronal activity (41). Local glucose metabolism and 18F-FDG brain uptake have been shown to be connected with the regional neuronal and synaptic activity due to neurotransmission and neurotransduction require a lot of energy (42). According to newer research, astrocytes also play a substantial role in the 18F-FDG-PET signal (43, 44).

**Figure 2:**
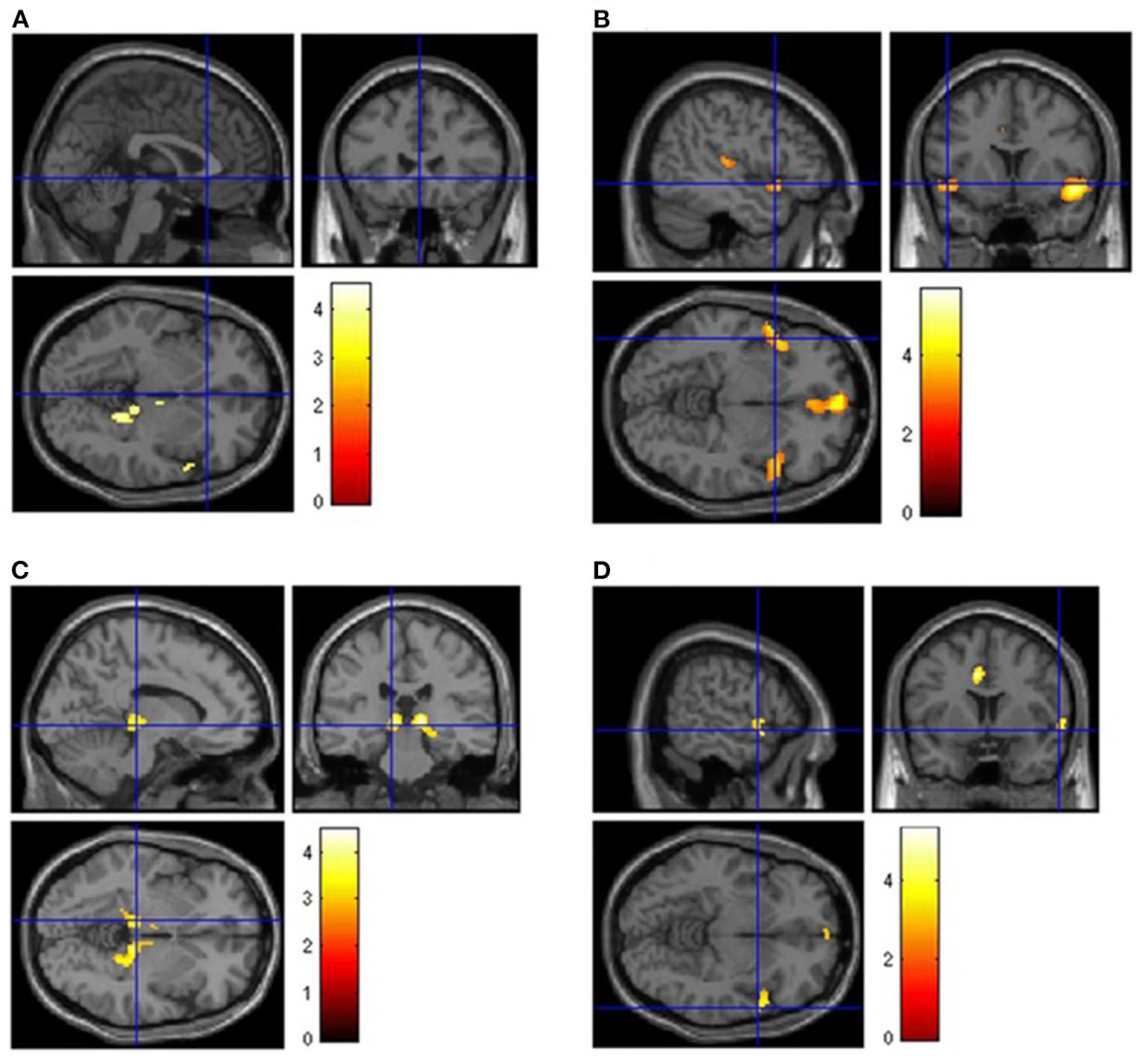
Brain [18F]FDG PET analysis. Regions of hypometabolism compared to controls in the 13 long COVID patients **(A)** and subgroups of patients showing persistence of anosmia **(B)**, fatigue **(C)**, or mild-to-moderate vessel [18F]FDG uptake **(D)**. Regions of significant difference are color-graded in terms of *Z*-values. Adapted from Sollini et al. (46) (License Number 5472490224310).

**Figure 3:**
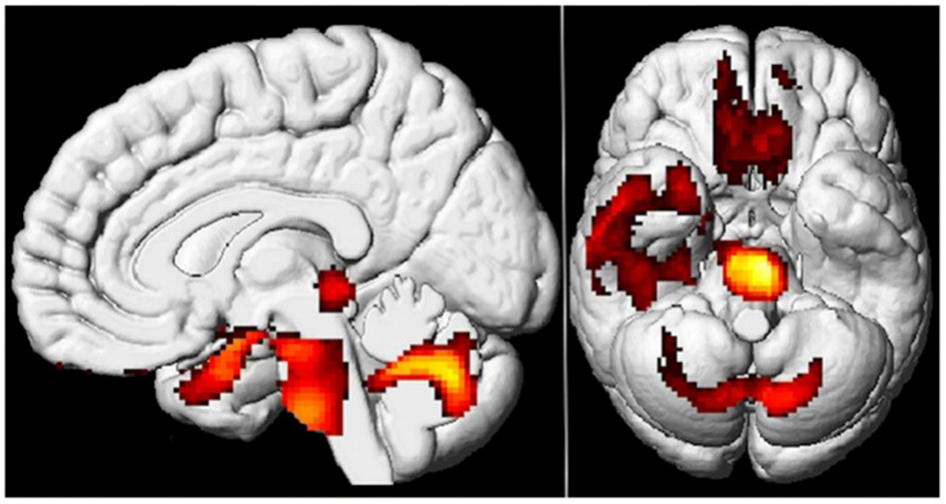
Brain 18F-FDG PET hypometabolism in patients with long COVID. In comparison to healthy subjects, the patients exhibit hypometabolism in the bilateral rectal/orbital gyrus, including the olfactory gyrus; the right temporal lobe, including the amygdala and the hippocampus, extending to the right thalamus; the bilateral pons/medulla brainstem; the bilateral cerebellum (*p*-voxel < 0.001 uncorrected, *p*-cluster < 0.05 FWE-corrected; SPM8 3D rendering). Adapted from Guedj et al. (45) (License Number 5474900644082).

According to certain studies, patients with Post COVID-19, who have chronic functional problems, have 18F-FDG-PET hypometabolism in several cerebrum areas (45–48). After analyzing the 18F-FDG cerebral PET of patients with PCNS, with a scientifically verified diagnosis of SARS-CoV-2 disease and chronic functional symptoms at least 3 weeks after the first infection, scientists discovered hypometabolism in: the bilateral orbital gyrus which contains the olfactory gyrus, the right parahippocampal gyrus, the right temporal lobe e.g., amygdala, hippocampus, and thalamus, the bilateral cerebellum and the bilateral pons/medulla brainstem (45–48) (Figures 2, 3). Furthermore, this hypometabolism was linked to the patients' problems e.g., memory and cognitive dysfunction, sleep disturbances and pain (45–47). Moreover, a new research with 18F-FDG-PET demonstrated the neurological long-term consequences of COVID-19 (49). The imaging revealed abnormal findings in more than 66% of the individuals, most of whom presented fronto-parietal hypometabolism (49). In conclusion, the 18F-FDG-PET data demonstrated above suggests that the frontal, temporal, and parietal lobes are sensitive to SARS-CoV-2 infection (45–49).

Interestingly, Verger et al. reported not only 18F-FDG-PET hypometabolism in cerebrum of patients with PCNS but also the demonstration of a brain impairment, and the differential diagnosis of PCNS in 18F-FDG-PET (47). The authors claims that an individual PET hypometabolism, suggesting network-based involvement, may indicate a real cerebrum impairment (47). Therefore, brain FDG PET could be a good type of imaging to objectify brain involvement in patients with PCNS—it might affect both a specific prognosis and treatment (47). Moreover, Verger et al. describe that PCNS should be differentiate from neurodegenerative diseases, encephalitis/encephalopathy, and psychiatric disorders in FDG PET (47). In addition, new PET radiotracers should be used in future studies to assess PCNS (47, 50, 51). For example, the translocator protein 18 kDa (TSPO) could be a good radiotracer for neuroinfammation PET targeting in PCNS because neuroinfammation is one of the primary hypotheses which explains cerebrum damage in patients with PCNS (47, 50, 51).

CT is one of the most frequently used imaging techniques in medicine due to: the short scan time, low cost and relatively high accuracy of an imaging examination in many disorders. In the case of PCNS imaging, CT is useful for detecting vascular alternations in the CNS (52–54).

Some cases in the literature of PCNS, in which CT was used, describe vascular changes in the brain. Patients with PCNS with lesions in CT imaging ranged from 53 to 85 years of age (52–54). The alternations in CT of CNS in most cases were not related to the clinical symptoms of PCNS (52–54). Typical manifestations of PCNS in CT imaging were: a moderate hypodensity in the frontal lobe (acute infarct), an insular area infarct, acute large parietal-occipital and cerebellar infarcts, acute infarcts of the middle cerebral arteries (MCAs), an occlusion and infarct of the posterior cerebral artery (PCA), a hemorrhagic stroke (48, 52–54). Moreover, CT angiography can make the changes visible such as: a thrombotic occlusion of a proximal M2 branch of the middle cerebral artery and an occlusion of the MCA (48, 52, 54). It should also be taken into account that some of these lesions may be related to age or chronic diseases of patients (52–54).

MRI is a non-invasive method that may give detailed, multi-parametric data on cerebrum structure, function, and metabolism (55). The lesions observed in MRI have a much wider scope of the PCNS imaging than PET/CT and CT (48, 56–58). They include, among others: vascular lesions in the CNS, changes in the brain, spinal cord and cranial nerves which confirm the diagnosis of GBS, neurodegeneration and gliosis (48, 56–58). Patients with PCNS with lesions in MR imaging ranged from 11 to 88 years of age (48, 56–58).

**Figure 4:**
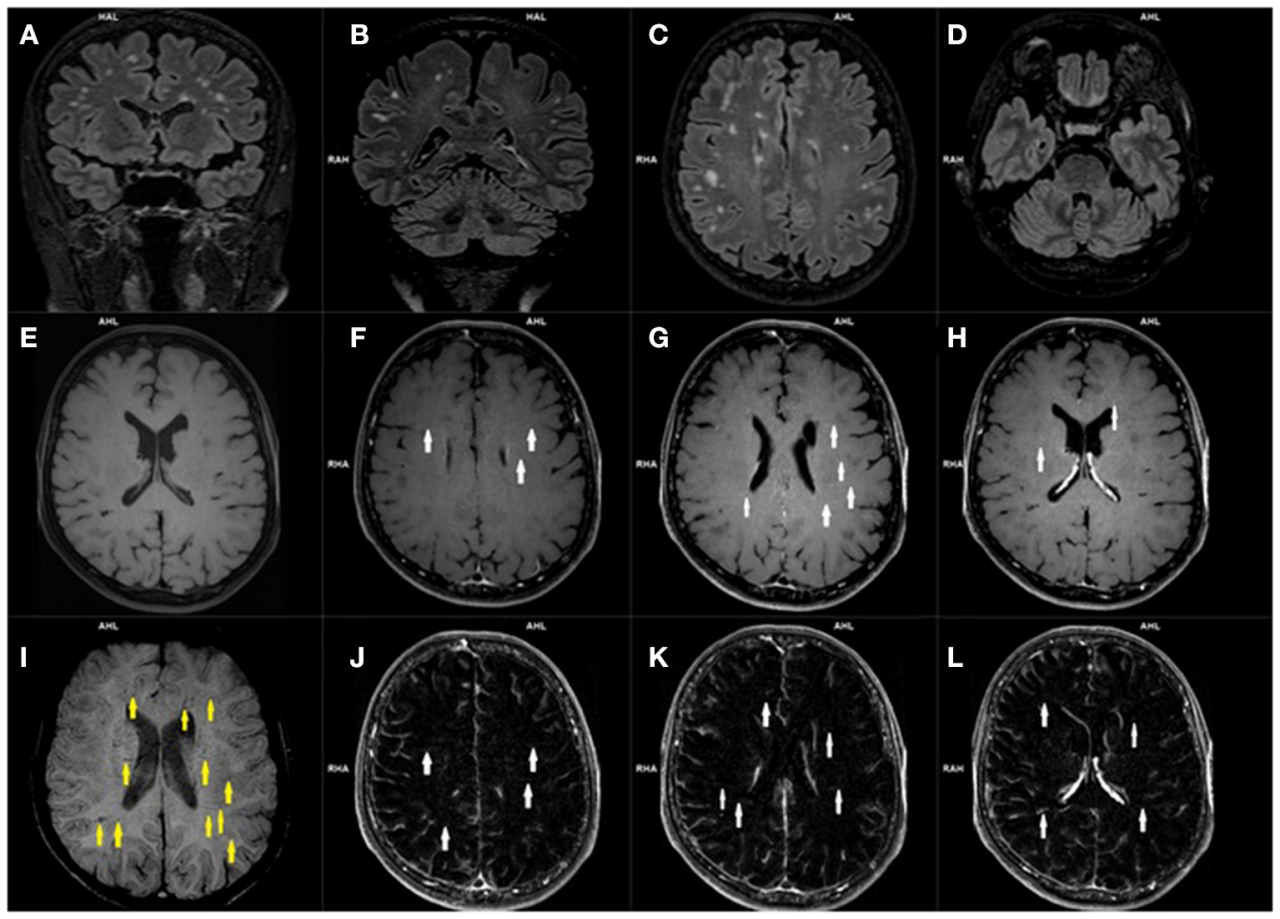
Magnetic resonance imaging of the brain of a patient with post COVID-19 symptoms. Coronal **(A, B)** and axial **(C, D)** 3D-FLAIR images demonstrate multiple hyperintensities located within subcortical and deep white matter. Axial precontrast T1-weighted image **(E)**, and contrast enhanced axial T1-weighted images **(E–H)** with axial T1-weighted subtraction maps **(J–L)** show subtle parenchymal enhancement along the course of deep located parenchymal veins (*white arrows*). Corresponding SWI image **(I)** demonstrates engorged some deep medullary veins (*yellow arrows*).

**Figure 5:**
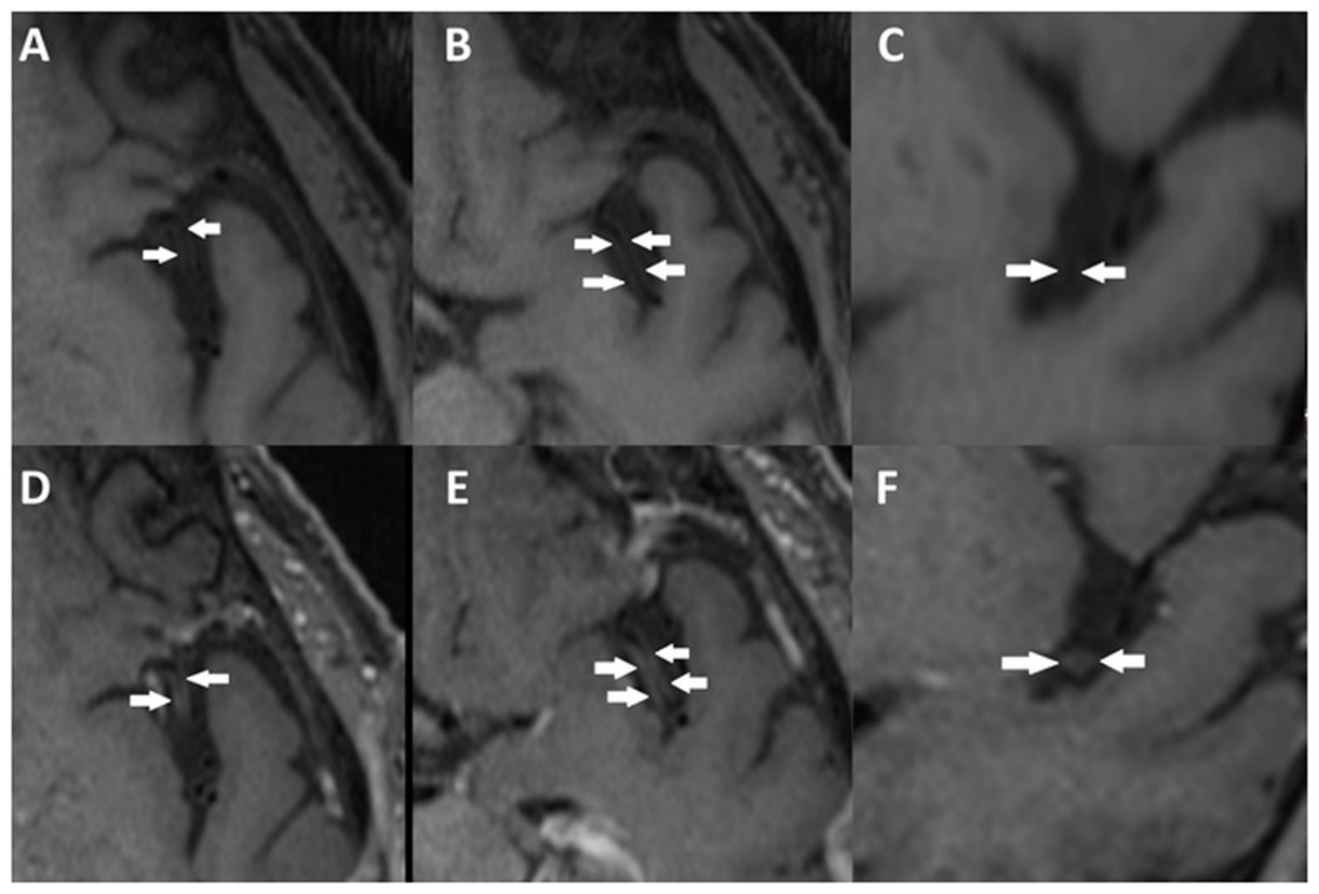
Vessel wall imaging (VWI-MR) of the brain of another patient who experienced post COVID-19 symptoms. Axial **(A, B)** and coronal **(C)** precontrast T1-weighted images, and contrast enhanced axial **(D, E)** and coronal **(F)** T1-weighted images, show segmental concentric wall thickening and enhancement of M2 left middle cerebral artery segments (*arrows*) consistent with cerebral vasculitis.

The cerebrovascular alternations in MRI of the CNS in most cases were not related to the neurological symptoms of PCNS (48, 56–58). There were some studies which describes patients with hyperintense subcortical images, as well as in occipital and frontal (bilaterally) white matter in T2-weighted and FLAIR sequences and hypointense lesions on T1-weighted images (48, 56–58). Typical vascular manifestations of PCNS in MR imaging were: an acute brain infarct in striatum, thalamus, pons, occipital lobes, temporal lobes and cerebellum, many small regions of restricted diffusion in the centrum semiovale which indicate a small acute infarction, an acute infarction near the frontal horn, blood vessels occlusion e.g., mild stenosis of the M1 segment, a thrombus in the basilar artery, bilateral P2 segment stenosis, intradural vertebral artery occlusion (48, 52, 53, 56–58). Interestingly, the occurrence of a cerebral vasculitis in the context of PCNS has also been described (48, 57, 58). Figure 4 shows exemplary hyperintensive foci and lesions in the form of engorgement of deep medullary veins and Figure 5 presents vasculitis changes in the form of thickening of the vessel wall, its irregularities and contrast enhancement (Figures 4, 5).

GBS has become increasingly frequent after the outbreak of pandemic (59–63). Several studies have shown a relationship between PCNS and GBS (59–63). Part of them describe lesions in the CNS observed in MRI (59–63). Alternations of the GBS in PCNS in the spinal cord demonstrated in MRI include an enhancement of the cauda equina nerve roots in T1-weighted images after gadolinium contrast (59–63). They are associated with the impairment in lower extremities (59–63). Furthermore, contrast-enhanced T1-weighted MR imaging of the head in GBS after COVID-19 revealed cranial nerves implication (more precisely enhancement of the bilateral facial nerves after contrast) (59–63). Approximately 18% patients have contrast-enhancement of the cranial nerves in GBS connected with the PCNS in MRI examination (59–63). Typically GBS after COVID-19 is associated with the: negative result of the examination for serum anti-glycolipid Ab (antibodies), a CSF test demonstrates albuminocytologic dissociation SARS-CoV-2 PCR in the CSF is negative (59).

**Figure 6:**
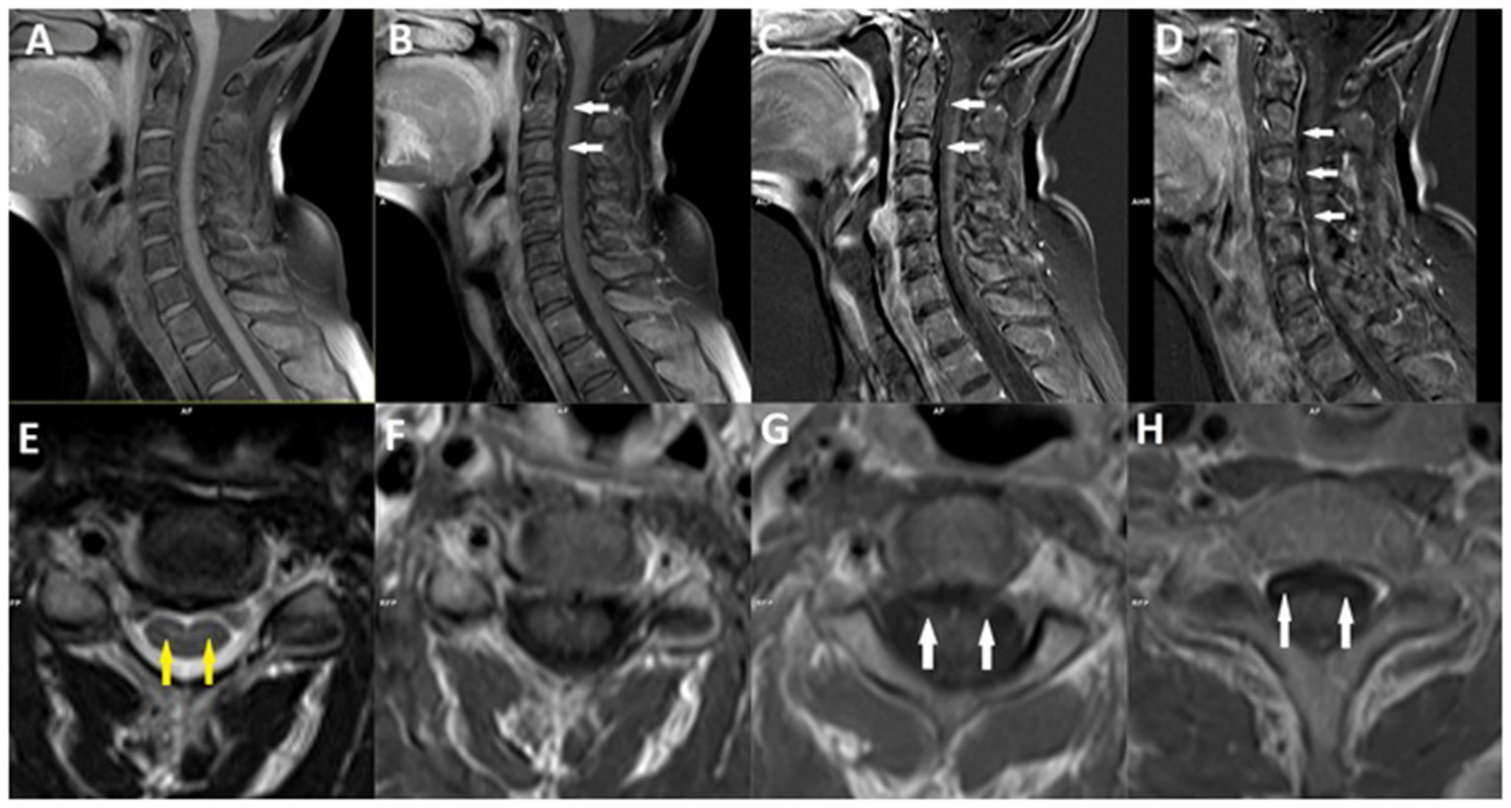
Magnetic resonance imaging of the cervical spine of a patient with chronic inflammatory demyelinating polyneuropathy (CIDP) after COVID-19 infection. Sagittal **(A)** and axial **(F)** precontrast T1-weighted images, and postcontrast sagittal **(B)** and axial **(G, H)** T1-weighted images with sagittal T1-weighted subtraction maps **(C, D)**, demonstrate bilateral enhancement of the ventral nerve roots (*white arrows*). Axial T2-weighted image **(E)** demonstrate discrete the hyperintensities in the anterior horns of gray matter (*yellow arrows*).

It is worth mentioning that GBS has the chronic counterpart involving peripheral nerves which called chronic inflammatory demyelinating polyneuropathy (CIDP) (64). What is more, some individuals may show an acute presentation of CIDP that strongly resembles GBS that it is sometimes difficult to differentiate them (65). A large number of studies describe the connection between COVID-19 and GBS, but some of them show an association between COVID-19 and CIDP (66). Thickening and enhancement of peripheral nerves which sometimes resemble onion bulbs, brachial and lumbosacral plexus, and nerve roots are characteristic MRI findings of CIDP (64–66). Furthermore, a third of individuals have cranial nerve involvement (64) (Figure 6).

Many hyperintense focal regions in periventricular and subcortical white matter, as well as semioval centers, might be identified in the cerebrum MRI after intravenous gadolinium contrast administration in long TR scans, particularly in FLAIR (67). These MRI results are linked to symptoms including persistent tiredness, headache, anxiety episodes, and severe depression which may be manifestations of the PCNS (67). These changes in the cerebral tissue are quite comparable to those found in ~40–45% of people experiencing migraine, systemic immunological disorders, and connective tissue inflammatory reactions (67). These disorders associated with the vasoconstriction, may cause microthrombosis which leads to neurodegeneration and gliosis (48, 67).

Furthermore, Douaud et al. investigated cerebrum lesions in MRI of patients with PCNS and discovered significant longitudinal effects (68). Many patients presented a reduction in gray matter thickness, in cerebrum size, and contrast of tissue in the orbitofrontal cortex and parahippocampal gyrus (68). Some of individuals demonstrated areas associated with the primary olfactory cortex in which changes in markers of tissue damage might be seen (68). These primarily brain imaging findings could be the features of a degenerative spread of the illness through olfactory pathways, of neurological inflammatory processes, or of the loss of sensory input (68). In addition, these examined patients with PCNS presented a cognitive decline (68).

Overall, PCNS should be considered a diagnosis of exclusion because there are currently no imaging findings considered to be specific for PCNS, and there is significant overlap with other neurologic illnesses (47, 69, 70). Therefore, clinical examination and EEG in conjunction with radiological examinations are also an important aspect to make a proper diagnosis of PCNS (47, 69, 70). The clinicians examining PCNS-like symptoms should remember about differential diagnoses e.g., stroke, cerebral vein thrombosis, neurodegenerative diseases, encephalitis, encephalopathy, seizures, insomnia, anxiety, depression, Post Traumatic Stress Disorder (PTSD), and other mental disorders, in which imaging techniques such as PET/CT, CT and MRI might be very helpful (47, 69, 70).

## Conclusions

In conclusion, PCNS is one of the most significant long-term worldwide public health problem that involves both hospitalized and non-hospitalized people. Age above 65, chronic pulmonary illness, heart diseases, high blood pressure, adiposity and diabetes are the most important risk agents for SARS-CoV-2 infection-related sequelae such as PCNS (71).

More studies are needed to define CNS neuroimaging in patients with PCNS. However, several imaging studies which describe lesions in PCNS may be of great clinical importance in the future. Several of them claim.

### PET/CT

The 18F-FDG-PET signal in the cerebrum is used as an index of activity of neurons. Researchers observed hypometabolism in the bilateral orbital gyrus, right parahippocampal gyrus, frontal lobes, parietal lobes, right temporal lobe, bilateral cerebellum, and bilateral pons/medulla brainstem after assessing the 18F-FDG cerebral PET of PCNS patients. Moreover, this hypometabolism has been connected to the individuals' disorders e.g., memory and cognitive failure, sleep disorders, and pain. Furthermore, 18F-FDG-PET is considered as a good type of imaging to differentiate PCNS from neurodegenerative diseases, encephalitis/encephalopathy, and psychiatric disorders.

### CT

CT is beneficial for identifying cerebrovascular lesions in the CNS in the PCNS imaging. In most patients, the changes in CT of the CNS were unrelated to the clinical manifestations of PCNS. An acute infarct of the frontal lobes, acute large parietal-occipital and cerebellar infarcts, acute infarcts of the MCA, an occlusion and infarct of the PCA, a hemorrhagic stroke, a blockage of a proximal M2 branch of the MCA, and an occlusion of the MCA were the most often manifestations of PCNS.

### MRI

Magnetic resonance imaging has a significantly broader spectrum of the PCNS imaging than PET/CT and CT. In MRI, typical signs of PCNS included: infarcts in the striatum, thalamus, pons, occipital lobes, termporal lobes, and cerebellum, many minor areas of restricted diffusion in the centrum semiovale, blood vessel occlusion, cerebral vasculitis, and a reduction in gray matter thickness, in brain size, and tissue contrast in the orbitofrontal cortex and parahippocampal gyrus. After contrast administration, several hyperintense focal areas in periventricular and subcortical white matter, and semioval centers, may be seen in cerebrum MRI in long TR scans. These MRI findings have been connected to manifestations that are similar to PCNS symptoms. Finally, changes in the GBS in the PCNS of the spinal cord shown in MRI include an enhancement of the cauda equina nerve roots in T1-weighted images with contrast. Additionally, contrast-enhanced T1-weighted MRI of the head in GBS, following COVID-19, demonstrated enhancement of the facial nerves. Furthermore, CIDP, as the chronic counterpart to GBS, associated with the Post COVID-19 condition presents thickening and enhancement of peripheral nerves, brachial and lumbosacral plexus, nerve roots and demonstrates the hyperintensities in the gray matter.

CT, PET/CT, and MRI examinations of the cerebrum should be performed in each case of PCNS (broad availability examinations), in order to assessment any abnormalities of the brain to better identify PCNS. Frequent usage and documentation of these imaging methods could accelerate the development of imaging techniques in PCNS. In summary, our review demonstrated that despite healing from an acute disease, the epidemic underlines the importance of continued, extensive follow-up of all patients with COVID-19, because they may have complications later such as PCNS.

## Author contributions

JO: material preparation, data collection, and writing-first draft of the manuscript. AG and BK: figures selection and description. AM-M: writing-first draft of the manuscript. All authors read and approved the final manuscript and agreed to be accountable for the content of the work.
